# Sleep quality and disturbances in adolescents and young adults with cancer compared to a healthy comparison group: results of the AYA-LE study

**DOI:** 10.1007/s00432-026-06627-1

**Published:** 2026-09-22

**Authors:** Carolin Wieland, Michael Friedrich, Annekathrin Sender, Katja Leuteritz, Hannah Brock, Kristina Geue, Anja Mehnert-Theuerkauf, Diana Richter

**Affiliations:** 1 Department of Medical Psychology and Medical Sociology, Comprehensive Cancer Center Central Germany (CCCG), University Medical Center Leipzig, Philipp-Rosenthal-Str. 55, 04103 Leipzig, Germany; 2 University Clinic for Psychosomatic Medicine and Psychotherapy, University Medical Center Magdeburg, Otto-von-Guericke Universität Magdeburg, Magdeburg, Germany

**Keywords:** AYA, Cancer, PSQI, Sleep, Psycho-oncology, Survivorship

## Abstract

**Purpose:**

Adolescents and young adults (AYAs) with cancer often exhibit riskier health behaviors, including poorer sleep patterns, compared to older individuals. Research on the relationship between cancer and sleep quality in AYA patients has yielded inconsistent results. This study investigates sleep quality in AYA patients compared to a healthy comparison group and examines associated sociodemographic, clinical, and psychosocial variables as well as physical activity.

**Methods:**

The prospective observational study included 407 AYA patients aged 18–39 years at diagnosis and 372 healthy participants aged 18–45 years, with data collected from May–September 2019. Sleep quality was assessed using the Pittsburgh Sleep Quality Index (PSQI). Validated questionnaires measured self-efficacy, social support, anxiety, depression, quality of life, lack of energy, and physical activity. Multiple regression analyses were employed to explore the variables associated with sleep quality.

**Results:**

The mean age of the AYA patients was 34.8 years (75.2% women) and 32.3 years of the healthy participants (61.8% women). AYA patients demonstrated significantly poorer subjective sleep quality (d=0.31), lower sleep efficiency (d=0.15), more frequent sleep disturbances (d=0.23), greater use of sleep medication (d=0.25), and higher daytime dysfunction (d=0.29). Anxiety, depression (HADS), quality of life (EORTC), and unmet needs for support through lack of energy (SCNS) were significantly associated with PSQI (adjusted R^2^=0.35).

**Conclusion:**

These findings underscore the relevance of sleep disturbances in AYA patients and support greater attention to sleep disturbances in survivorship care. Routine assessment of sleep quality may help identify AYA patients who could benefit from further evaluation and appropriate support.

## Introduction

A recent report by researchers at the International Agency for Research on Cancer (IARC) and the American Cancer Society (ACS) indicated that in 2024 approximately 21 million new cases of cancer would be diagnosed worldwide, with approximately 10 million cancer-related deaths. It is expected that the annual number of new cancer cases will reach 34 million by 2050, representing a 67% increase compared to 2024 (Sung et al. 2026). In Germany, approximately 17,000 adolescents and young adults (AYA) between the ages of 15 and 39 are diagnosed with cancer annually, representing nearly 3% of all new cancer cases (Robert Koch Institute 2025). Although AYA patients have a relatively high five-year survival rate of 83–86%, largely due to advances in cancer therapies (Miller et al. 2020; Janssen et al. 2021) they often face numerous long-term physical and psychological challenges.

The care of AYA patients represents a particular challenge for the clinical staff, since the needs and the living situation at this age group differ significantly from those of children and older cancer survivors. Young adulthood is characterized by complex cognitive and psychosocial changes, such as financial independence, career development, or family planning (Jin et al. 2021). Cancer-related challenges, including alterations in physical appearance (Moore et al. 2021), potential loss of reproductive ability, and psychosocial limitations, can be highly disabling for young adults (Stroeken et al. 2024). In addition, treatment-related side effects, such as tiredness, dyspnea and weight loss, can lead to severe psychological stress (Miller et al. 2016). Elevated anxiety and depression scores in AYA patients indicated a significant unmet need for psycho-oncological care (Osmani et al. 2023).

AYA patients are at an elevated biological and behavioral risk for circadian dysregulation and short sleep due to the shift in circadian rhythm and self-selected bedtime that occurs during adolescence and young adulthood (Hagenauer et al. 2009; Crowley et al. 2014). The occurrence of sleep disturbances during cancer treatment can be attributed to a number of factors, including the presence of physical symptoms, the side effects of medication and the necessity of hospitalization (Walter et al. 2015).

Sleep disturbances occur throughout the course of cancer treatment and beyond in many AYAs and may persist as patients move into older adulthood (Olson 2014; Ameringer et al. 2015; Daniel et al. 2017). The research on this subject is scant and inconsistent in its findings. Daniel et al. (2016) indicated no significant differences in the amount or quality of sleep between AYA cancer survivors (16–30 years) and healthy controls of the same age. In contrast, Garland et al. (2023) showed that 52% of young adults (YA) cancer survivors had poor sleep quality. For example, 56% of YA cancer survivors reported prolonged sleep onset delay (> 30 min), 33% slept <7 h per night, and 55% described habitual sleep efficiency of <85%. Over 80% of teenagers and young adults (TYA) cancer patients and more than half of TYA cancer survivors (13–24 years) reported poor sleep quality with a PSQI score >5 (Fortmann et al. 2021). Sleep difficulties emerged as one of the three most common symptoms among AYA patients (12–25 years) (Steineck et al. 2022).

Sleep disturbances in AYA patients correlate with psychological distress (Luo et al. 2024) and pain (Savard et al. 2015), the pain is associated with an increased cortisol level (Stehlik et al. 2018). A strong connection exists between sleep and immune function, meaning that sleep disturbances can affect resistance to infectious diseases and increase the risk of cancer and mortality rates (Irwin 2015). In a study of Garland et al. (2023), around 45% of YA cancer survivors (15–39 years) reported taking sleeping pills, with around 20% using them three or more times a week. YA cancer survivors with severe mental health problems were 2.4 times more likely to take sleeping pills.

So far, there have only been a few studies on the health behavior of AYA patients (Warner et al. 2016; Stroske et al. 2021; McGrady et al. 2024). Especially on the issue of sleep quality, existing research findings are inconsistent in various ways and there is a lack of data for sufficient support in further treatment. For the clinical treatment of AYA patients, it is important to recognize the risk for sleep disturbances and to understand the relationship between sleep quality and physical and mental health. Therefore, the aim of this study was to examine the sleep quality of AYA patients in more detail, as the health behavior of AYA patients plays a significant role in determining the further course of the disease and the process of aftercare. In particular, the following questions were examined:

- What are the differences in sleep quality between AYA patients and a healthy comparison group?
- Which sociodemographic, clinical, and psychosocial variables, as well as physical activity, are associated with sleep quality in AYA patients?

## Materials and methods

### Study participants

The sample is based on the prospective longitudinal AYA-LE study (Life situation and psychosocial care of adolescent and young adult cancer survivors—AYA Leipzig study), which investigated the psychosocial life situation (e.g. quality of life) of AYA patients (Geue et al. 2021). The data were collected from May 2014–September 2021 throughout Germany with six measurement time points (t1: after acute treatment from May 2014–December 2015, t2: 12 months after t1, t3: 30 months after t2, t4: 12 months after t3) examining different issues of the respondents’ life situation. We used data generated by the fourth survey (t4: May 2019–September 2019), which focuses on AYA patients’ health behaviors, including sleep quality. Study participants were recruited from 16 acute oncology hospitals, four rehabilitation clinics and two tumor registries. AYA patients between the ages of 18–39 years with a first manifestation of a malignant cancer within the previous four years were included. AYA patients could register online or by telephone. The survey was implemented using an online questionnaire or, alternatively, a hard copy version posted to participants (Leuteritz et al. 2017). A sample with the data from the second survey (t2: January 2019) of healthy young adults aged 18–45 years from Leipzig, who were contacted via the Leipzig residents’ registration office, served as a healthy comparison group. The healthy comparison group completed the questionnaire in paper format. Subjects were excluded from the respective sample, if they were physically or cognitively unable to participate, did not have a sufficient level of German or had not given informed consent. All study participants received a compensation fee of 10€ for completing the questionnaire. The study was approved by the ethics committee of the University of Leipzig (November 3, 2017, file number: 436-17-131 12017).

### Measures

The survey included sociodemographic and clinical data as well as several standardized questionnaires. Sociodemographic data included gender, age at interview, marital status, partnership, and living situation. The AYA patients also provided clinical data: diagnosis, time since diagnosis, age at diagnosis, and days of sick leave in the year 2018. All data on sociodemographic and clinical topics were based on self-reported information.

### Pittsburgh sleep quality index (PSQI)

The Pittsburgh Sleep Quality Index (PSQI) is a self-rated questionnaire that measures sleep quality and disturbance over a one-month period. The questionnaire consists of 19 items, which comprise seven components: subjective sleep quality, sleep latency, sleep duration, sleep efficiency, sleep disturbances, use of sleep medication and daytime dysfunction (Buysse et al. 1989). The sum of the ratings on a four-point Likert scale from 0 “not at all” to 3 “three or more times per week” result in a PSQI global score (range 0–21); a PSQI global score >5 indicates poor sleep quality. In the original validation paper, the PSQI had an internal consistency and a reliability coefficient (Cronbach’s alpha) of α=0.83 for its seven components (Buysse et al. 1989; Backhaus et al. 2002).

### General self-efficacy short scale (ASKU)

The General Self-Efficacy Short Scale with three items assesses optimistic self-beliefs to cope with a variety of daily hazards and difficulties in life. The response categories on the five-point Likert scale range from 1 “not at all true” to 5 “completely true”. A mean ASKU score is calculated from the sum of the points. The test has good reliability (McDonalds ꞷ=0.81–0.86) and validity (Beierlein et al. 2013).

### Social support questionnaire (F-SozU)

The questionnaire on social support measures the perceived and anticipated social support (Hermann 2021). The internal consistency is 0.94. The short version has 14 items (K-14) that are rated on a five-point Likert scale from 1 “does not apply at all” to 5 “applies exactly”. The total score is calculated based on the mean scores for all items. Greater perceived and expected social support is indicated by higher scores (Fydrich et al. 2009).

### Hospital anxiety and depression scale (HADS)

The Hospital Anxiety and Depression Scale was used to assess anxious and depressive symptoms (Herrmann-Lingen et al. 1995). The objective and content-valid Questionnaire consists of 14 items on a four-point Likert scale (range 0–3) measured on the subscales anxiety and depression. The original questionnaire defined three ranges for the subscales: 0–7 (unremarkable), 8–10 (suspicious), and 11–21 (noticeable). High scores implicate high values of anxiety and depression (Bjelland et al. 2002).

### European organization for the research and treatment of cancer quality of life questionnaire-core 30 (EORTC QLQ-C30)

The EORTC QLQ-C30 questionnaire assesses health-related quality of life (HRQoL) in cancer patients and consists of 28 items measured on a four-point Likert scale (range 0–4) and two global health/ QoL-Items with seven response options (range 1–7). Scoring is on a 0–100 scale. It includes five functional scales, three multi-item symptom scales, six single-item symptom scales, and a two-item global health and quality of life scale. Higher scores on the functional subscales and global health indicate better health, whereas higher scores on the symptom subscales indicate higher symptom burden. There is acceptable internal consistency of Cronbach’s α >0.70 for all scales (with exception of role functioning) (Aaronson et al. 1993). For our study, we used the functional quality of life score of the functional subscales (physical, role, cognitive, emotional, and social function).

### Supportive care needs survey questionnaire (SCNS)

The established 34-item Supportive Care Needs Survey-Short Form (SCNS-SF34G) self-report questionnaire identifies the nature and extent of perceived or unmet care needs of cancer patients. The questionnaire has a high reliability (Cronbach’s alpha range of 0.82–0.94) and represents construct and content validity for the German version. The SCNS-SF34G contains five components: psychological, physical and daily living, health system, treatment and sexuality. Patients indicated their need for support on a five-point scale (1=no need, not applicable; 2=no need, already supported; 3=low need; 4=moderate need; 5=high need). The total score ranged from 0–100, with a higher score indicating a higher level of neediness (Lehmann et al. 2012). For our study, we analyzed the item “Lack of energy and tiredness” to check whether there was a need for support.

### Exercise and sports activity questionnaire (BSA-F)

The screening instrument for examining daily physical and sporting activity in the last four weeks is a questionnaire that examines physical activity at work in three different areas on a four-point Likert scale with three items from 0 “none” to 3 “very”, as well as activity in leisure time with nine items (“number of days”/“how long”/“not done”) and sporting activity with one item (“yes”/“no”). For each activity, the three dimensions of frequency, duration and type were taken into account. For the total score, the frequency and duration of each type of exercise were multiplied and the sum was calculated, which when divided by four gave the amount of physical activity (in minutes per week). Higher scores reflect increased physical activity. The instrument has high construct and criterion validity (Fuchs et al. 2015).

### Statistical analyses

The statistical analyses were performed using IBM SPSS Statistics 26 (IBM Corp. 2019). Missing values were estimated at item level using the Expectation Maximization (EM) algorithm implemented in SPSS. The assumed values that exceeded the possible range were set to the next possible value. AYA patients and healthy comparison group were compared for sociodemographic characteristics using descriptive statistics (mean, standard deviation, frequencies), t-test (significance of means), and χ^2^-test. A significance level of 0.05 was set for all tests, and the *p*-values were two-tailed. To estimate the effect sizes d according to Cohen’s d (0.2 small effect, 0.5 medium effect and 0.8 large effect), the mean differences between the patient and healthy comparison group (corresponding to the pooled standard deviation) were assessed (Cohen 1988). Two-tailed t-tests for independent samples were performed to determine differences in sleep quality components and PSQI global score between the AYA patients and the healthy comparison group. A multiple linear regression analysis with backward elimination was applied to identify variables independently associated with AYA patients’ sleep quality. The independent variables were tested for intercorrelations above r >0.80 to avoid multicollinearity and associated standard errors. Following variables were included as independent factors:

Sociodemographic variables:

- Gender (female/male), age at interview (years), partnership (no/yes);

Clinical variables:

- Diagnosis (solid/non-solid), time since diagnosis (months), days of sick leave (≤ />9 days);

Psychosocial variables:

- Self-efficacy (ASKU), perceived social support (F-SozU), anxiety and depression total scale (HADS), functional quality of life total score (EORTC) and the item of the supportive care needs survey questionnaire “unmet need-lack of energy and tiredness” (SCNS);

and health behavior variable:

- Physical activity (BSA-F)

All variables were selected for inclusion in the multiple linear regression model, as these factors were known to affect sleep quality in AYA patients based on existing research (Daniel et al. 2016; Garland et al. 2023).

## Results

### Sample

At the fourth survey measurement point (t4), n=407 AYA patients and n=372 healthy participants took part in the study (Table 1). The mean age of the AYA patients was M=34.8 years (SD=6.3) and of the healthy comparison group M=32.3 years (SD=5.8). Women formed the majority in both samples, but the proportion of women among the AYA patients was slightly higher (75.2% vs. 61.8%). The AYA patients, who were on average around two years older than the participants in the healthy comparison group, were more likely to be married or divorced, in a similar proportion to the participants in the healthy comparison group in a partnership, and more often living alone or with their parents. With regard to the target variables, statistically significant between-group differences were observed for physical activity (BSA-F), self-efficacy (ASKU), and social support (F-SozU) (all *p* < 0.05); however, the corresponding effect sizes were small (Cohen’s d = −0.17, −0.15, and −0.18, respectively). AYA patients also reported significantly lower functional quality of life, with a significantly larger effect size (d = −0.75).

**Table 1:** Sociodemographic, clinical, and psychosocial characteristics and health behavior (physical activity)

Variable	AYA patients^1^	Healthy comparison group^1^	Group differences	
	*n*=407 (t4)	*n*=372	*P*^2^	Cohen’s *d/*Cramer’s V
*Sociodemographic variables*				
Gender*, n *(%)			<.001	0.14
Female	306 (75.2)	230 (61.8)		
Male	101 (24.8)	142 (38.2)		
Age at interview, years, *M (SD)*	34.8 (6.3)	32.3 (5.8)	<.001	
Range in years (Min–Max)	22.5–45.9	20.1–43.6		
Marital status, *n (%)*^1^			<.001	0.15
Single	219 (53.8)	253 (68.0)		
Married (living together)	168 (41.3)	103 (27.7)		
Married (separated)	4 (1.0)	5 (1.3)		
Divorced	14 (3.4)	10 (2.7)		
Partnership, yes, *n* (%)	324 (79.6)	287 (77.2)	.483	0.03
Living situation, *n* (%)			<.001	0.14
Alone	104 (25.6)	76 (20.7)		
With a partner	267 (65.6)	244 (66.3)		
Shared apartment	17 (4.2)	39 (10.6)		
With parents	19 (4.7)	9 (2.5)		
*Clinical variables*				
Diagnosis, *n *(%)				
Solid cancer	277 (68.1)			
Non-solid cancer	130 (31.9)			
Time since diagnosis, months, *M (SD)*	62.3 (9.8)			
Range (Min–Max)	45.5–98.3			
Age at diagnosis, years,* M (SD)*	29.6 (6.2)			
Range *(Min–Max)*	18.0–39.9			
Days of sick leave in the year 2018, *n *(%)				
≤9 days	264 (64.9)			
>9 days	143 (35.1)			
*Psychosocial variables*				
Self-efficacy, *M (SD)*	4.1 (0.7)	4.2 (0.6)	<.05	− 0.15
Social support, *M (SD)*	4.4 (0.7)	4.5 (0.6)	<.05	− 0.18
Anxiety and depression, *M (SD)*	10.9 (7.2)			
Functional quality of life, *M (SD)*	71.4 (22.2)	85.5 (13.9)	<.001	− 0.75
Unmet need-lack of energy, *n *(%)	190 (46.7)			
*Health behavior variable*				
Physical activity, min/week, *M (SD)*	571.7 (762.4)	723.3 (973.8)	<.05	− 0.17

M, mean; SD, standard deviation; ^1^ = N and percentages may not add up to 100 due to missing data

^2^ = based on t-tests for independent groups

### Comparisons of sleep quality between AYA patients and the healthy comparison group

The mean PSQI global score was M=6.3 (SD = 3.4) in AYA patients and 5.5 (SD = 2.9) in the healthy comparison group. The groups differed significantly in PSQI global score (*p* < 0.001; Table 2). Using the established PSQI cut-off of >5, 51.1% of AYA patients and 40.1% of the healthy comparison group were classified as having poor sleep quality (χ^2^ test: *p* < 0.05). In addition, AYA patients reported significantly poorer subjective sleep quality (d = 0.31), lower sleep efficiency (d = 0.15), more frequent sleep disturbances (d = 0.23), greater use of sleep medication (d = 0.25), and higher daytime dysfunction (d = 0.29) than the healthy comparison group. No significant group differences were found for sleep latency or sleep duration.

**Table 2:** Comparison of the Pittsburgh sleep quality index (PSQI) components and global score between the two samples

	AYA patients	Healthy comparison group	t-value	*p*-value	Cohen’s *d*
	*M (SD)*	*M (SD)*			
Sleep quality [0–3]	1.3 ± 0.7	1.1 ± 0.7	4.283	**<.001**	0.31
Sleep latency [0–3]	1.2 ± 1.0	1.2 ± 0.9	0.664	.507	0.05
Sleep duration [0–3]	0.6 ± 0.8	0.6 ± 0.8	0.929	.353	0.07
Sleep efficiency [0–3]	0.7 ± 1.0	0.6 ± 0.9	2.007	**.045**	0.15
Sleep disturbances [0–3]	1.1 ± 0.5	1.0 ± 0.4	3.178	**.002**	0.23
Use of sleep medication [0–3]	0.2 ± 0.6	0.1 ± 0.3	3.555	**<.001**	0.25
Daytime dysfunction [0–3]	1.1 ± 0.8	0.9 ± 0.7	4.086	**<.001**	0.29
PSQI global score [0–21]	6.3 ± 3.4	5.5 ± 2.9	3.933	**<.001**	0.28

For each measure higher scores indicate poorer sleep quality Note. Bold p-values indicate statistically significant differences between AYA patients and the healthy comparison group (p < .05)

M, mean; SD, standard deviation

Among the reasons for poor sleep quality, 37.1% of the 407 AYA patients experienced increased sleep latency. Additionally, 51.6% woke up during the night or early morning, while 45.2% reported waking to use the toilet. Breathing difficulties affected 3.2% of AYA patients, and 5.9% experienced loud coughing or snoring. Furthermore, 8.9% reported being too cold, whereas 24.6% felt too warm. Furthermore, 19.4% of AYA patients experienced pain at least once per week, and 11.6% reported bad dreams at least once per week.

The AYA patients’ time to fall asleep (M=25.2 min, SD=29.9) was similar to the healthy comparison group (M=24.1 min, SD=24.1). 16.5% of the AYA patients and 17.3% of the healthy comparison group needed more than 30 min to fall asleep. 45.2% of the AYA patients and 44.3% of the healthy comparison group slept more than 8 h. AYA patients (M=6.6 h, SD=1.2) slept on average about the same amount as the healthy comparison group (M=6.8 h, SD=1.1). 4.4% of the AYA patients and 1.1% of the healthy comparison group took sleeping pills once or several times a week. The majority (64.6%) of the AYA patients sleep together with their partner in a bed. These partners noted loud snoring in 27.7% long pauses in breathing during sleep in 4.2%, twitching or jerky leg movements in 24.1%, nighttime periods of confusion or disorientation in 3.5%, or other forms of restlessness at least once or more per week during sleep in 17.8% of the AYA patients.

### Variables associated with sleep quality in AYA patients

Multiple linear regression models with backward elimination were used to identify factors associated with sleep quality among AYA patients (Table 3). In the final model of multivariable linear regression with stepwise backward regression, anxiety and depression (b=0.10, *p*<0.01), the functional quality of life (b=− 0.04, *p*<0.001) and need for support due a lack of energy and tiredness (b=1.23, *p*<0.001) were significantly associated with lower sleep quality. The final model explained 36% of the variance (R^2^=0.36). The remaining sociodemographic (gender, age at interview, partnership), clinical (diagnosis, time since diagnosis, days of sick leave), psychosocial variables (self-efficacy, social support) and the health behavior variable (physical activity) were not significantly associated with sleep quality in the final model.

**Table 3:** Models of backward stepwise regression analysis of variables associated with Pittsburgh sleep quality index global score in AYA patients

		Model 1 with all independent variables			Final model after backward elimination	
Variable	*B *(SE)	95%CI	*Beta*	*B* (SE)	95%CI	*Beta*
*Sociodemographic*						
Gender (0=male, 1=female)	0.15 (0.34)	− 0.51; 0.81	.02			
Age at interview (years)	0.01 (0.02)	− 0.04; 0.06	.03			
Partnership (0=no, 1=yes)	− 0.37 (0.35)	− 1.05; 0.31	− .05			
*Clinical*						
Diagnosis (0=solid, 1=non-solid)	0.19 (0.33)	− 0.45; 0.83	.03			
Time since diagnosis (months)	0.01 (0.01)	− 0.03; 0.03	.01			
Days of sick leave (≤/>9 days)	0.32 (0.29)	− 0.25; 0.89	.05			
*Psychosocial*						
Self-efficacy (ASKU)	− 0.08 (0.25)	− 0.57; 0.42	− .02			
Social support (F-SozU)	− 0.16 (0.24)	− 0.63; 0.31	− .03			
Anxiety and depression (HADS)	0.09 (0.03)	0.02; 0.15	.18^**^	0.10 (0.03)	0.04; 0.16	.21^**^
Functional quality of life (EORTC)	− 0.04 (0.01)	− 0.06; − 0.02	− .27^***^	− 0.04 (0.01)	− 0.06; − 0.02	− .28^***^
Unmet need-lack of energy (SCNS)	1.22 (0.34)	0.55; 1.88	.18^***^	1.23 (0.33)	0.57; 1.88	.18^***^
*Health behavior*						
Physical activity (BSA-F)	0.00 (0.00)	0.00; 0.00	.01			
*R*^*2*^		0.36			0.36	
Adjusted *R*^*2*^		0.35			0.35	
*F* for change in *R*^*2*^		18.75			1.26	

*p<0.05; **p<0.01; ***p<0.001

## Discussion

The present study provides further evidence that sleep disturbances are relevant among AYA patients. Compared with the healthy comparison group, AYA patients reported poorer overall sleep quality, although the observed differences were generally small. Within the AYA sample, poorer sleep quality was associated with higher anxiety and depression, lower functional quality of life, and unmet needs related to lack of energy and tiredness.

### Comparison of sleep quality between AYA patients and the healthy comparison group

One aim of this study was to explore differences in sleep quality between AYA patients and a healthy comparison group. Poor sleep quality (PSQI > 5) was common in both groups, affecting 51.1% of AYA patients and 40.1% of the healthy comparison group. In addition, AYA patients had significantly higher PSQI global scores than the healthy comparison group. However, compared to the healthy comparison group, the AYA patients had significantly poorer sleep quality. This finding aligns with recent research by Garland et al. (2023), which reported that 52% of YA cancer survivors had poor sleep quality. The discrepancy between our results and those of Daniel et al. (2016), who found no significant differences in sleep quality between AYA cancer survivors and healthy AYA controls may be attributed to methodological variations or changes in sleep patterns over time post-treatment. In another study by Daniel et al. (2017), 48% of AYA cancer survivors (12–25 years) and in the study by Fortmann et al. (2021), 84% of TYA cancer patients and 63% of TYA cancer survivors (13–24 years) had an elevated PSQI >5. Vaughan et al. (2022) showed in their study, that over 50% of AYAs with cancer (15–25 years) reported sleep problems. Daniel et al. (2017) indicated increased sleep disturbances in AYAs (12–25 years), with prolonged time to fall asleep (41%) and reduced sleep efficiency (31%). In the literature, a higher consumption of sleeping medication was investigated in 44% of YA cancer survivors (15–39 years), with YA cancer survivors with psychological stress taking sleeping medication more frequently (Garland et al. 2023).

Furthermore, circadian and hormonal changes as well as external factors such as work and social commitments also contribute to later bedtimes and shorter sleep duration (Hagenauer et al. 2009; Crowley et al. 2014). Additionally, the side effects of cancer treatment, such as chemotherapy and radiation treatment-related late effects such as urinary incontinence, night sweats and gastrointestinal symptoms, may also be causal factors (Savard et al. 2015). After completing cancer treatment, AYAs with cancer face additional stressors, including fear of recurrence (Lane et al. 2019), stress of returning to school and/or work, and treatment decisions (Perales et al. 2016). Some pain medications, such as opioids, can have sedative effects (Stein 2018) as well as affecting sleep architecture (Cao and Javaheri 2018), which can promote poor sleep.

### Impact of sociodemographic, clinical, and psychosocial variables and health behavior (physical activity) on the sleep quality of AYA patients

Our research identified a significant association between PSQI global score and anxiety and depression levels (HADS). This aligns with prior studies showing poorer sleep quality and higher rates of anxiety and depression among younger cancer survivors (Champion et al. 2014). Sleep disturbances may both result from and exacerbate psychological distress (Luo et al. 2024), consistent with a potentially bidirectional relationship between sleep quality and mental health. Neuroendocrine and immunological mechanisms could also intensify this link in cancer survivors (Miller et al. 2008). These findings highlight the close relationship between sleep quality and psychological distress and suggest that both domains should be considered when assessing supportive care needs in AYA patients.

A significant relationship was observed between PSQI global score and functional quality of life (EORTC). Sleep disturbances may arise from physical symptoms like nocturnal pain or cognitive issues manifesting as rumination. Additionally, social and role-related stressors—such as starting a career or family—may further exacerbate sleep disturbances.

The PSQI showed significant association with unmet supportive care needs (SCNS), particularly regarding lack of energy and tiredness. Previous research highlights the persistent need for medical and psycho-oncological support among AYA patients, even years after treatment (Geue et al. 2014; Smrke et al. 2020). The association between unmet needs related to lack of energy and tiredness and poorer sleep quality highlights the importance of considering fatigue- and energy-related concerns when assessing sleep disturbances in AYA patients.

These findings support the consideration of sleep disturbances within outpatient and survivorship care for AYA patients. The observed association between sleep quality and psychological distress suggests that anxiety and depression should be considered when evaluating sleep-related concerns. Further research is needed to determine whether interventions targeting psychological distress can also improve sleep outcomes and reduce supportive care needs.

The sociodemographic characteristics gender, age, and partnership could not explain a substantial amount of variance in the PSQI global score. Previous research indicates that younger cancer patients and women are at higher risk for sleep disturbances (Peersmann et al. 2022b). Participants were predominantly women (75%), had a mean age of approximately 35 years, and most were living in a partnership. The resulting limited heterogeneity in some characteristics may have reduced the ability to detect associations reported in more diverse AYA populations.

The majority of the AYA patients surveyed had a solid cancer and the minority had a haematological cancer. However, the variable diagnosis showed no significant effect on sleep quality in our study. Previous studies stated that adolescent and young adult childhood cancer patients (12–26 years) with hematological cancer show a higher risk of sleep disturbance (Peersmann et al. 2022a). Time since diagnosis was not significantly associated with sleep quality in our study. This is broadly consistent with Garland et al. (2023), who identified psychological distress and poorer mental and physical functioning as important correlates of poor sleep quality among YA cancer survivors.

Within the AYA sample, psychosocial variables such as self-efficacy (ASKU) and social support (F-SozU), as well as the health behavior variable physical activity (BSA-F) were not significantly associated with sleep quality in our study. Research suggests that regular physical activity enhances sleep quality, whereas reduced activity may impair it (Chen et al. 2017; Ferreira et al. 2023). Higher self-efficacy has been linked to better management of disease-related symptoms, including improved sleep quality (Li et al. 2024).

The absence of significant associations should be interpreted cautiously. It may partly reflect characteristics of the study sample and limited variability in some of these factors.

### Study limitations

Although the data were derived from a longitudinal study, the present analyses were cross-sectional and based solely on data collected at the fourth assessment. Consequently, temporal and causal relationships between the examined variables cannot be established. For example, the analysis does not take into account potential changes in sleep quality over time, namely whether anxiety and depression lead to poor sleep quality or vice versa. Longitudinal analyses across multiple assessment points would be needed to capture changes in sleep quality over time and to clarify the temporal relationships between sleep disturbances and associated variables.

Furthermore, as the present analyses included participants who took part in the fourth assessment of the longitudinal study, selective participation and attrition across previous study waves cannot be excluded. Participants may therefore represent a selected subgroup of the original cohort. This potential selectivity of the sample may partly explain why some associations reported in previous studies were not observed in the present study.

Participants might overestimate or underestimate their sleep quality or psychosocial stress. The study relied solely on subjective measures of sleep quality, such as the PSQI, without incorporating objective sleep assessments like actigraphy or polysomnography. The lack of objective sleep measures limits the ability to complement and validate the self-reported sleep disturbances.

The representativeness of the study sample should be considered when interpreting the findings. The majority of participants were women (75%), potentially limiting the generalizability of our findings, particularly to male AYA patients. A predominance of female participants has also been reported in other AYA studies (Buro et al. 2023; Schulte et al. 2021). In addition, participants had a mean age of approximately 35 years. Differences in age distribution may partly contribute to discrepancies between our findings and those of previous studies that included younger AYA populations (Daniel et al. 2017; Peersmann et al. 2022a, 2022b). The mean time since diagnosis was approximately 62 months, indicating that our findings primarily reflect the experiences of longer-term AYA patients and may not be fully generalizable to AYA patients at earlier stages following diagnosis.

Furthermore, while multiple variables were analyzed, other potentially relevant variables, such as socioeconomic status, comorbid conditions, and the use of sleep-promoting strategies, including relaxation or mind–body techniques, were not assessed or fully accounted for.

### Implications for research and practice

From a clinical perspective, our findings support greater attention to sleep disturbances in AYA patients during and after treatment and suggest that routine assessment of sleep quality may be useful.

Such assessments could encompass questions regarding the presence and impact of sleep disturbances as well as the need for support. Further research is needed to establish clinically meaningful thresholds and guidance for when sleep disturbances in AYA patients warrant further assessment or intervention. The PSQI is a subjective measurement tool, thus objective sleep measurements, such as polysomnography and actigraphy, would be useful in further research to complement our findings.

Given the observed association between poor sleep quality and psychological distress, psycho-oncological support may be particularly relevant for AYA patients experiencing sleep disturbances. Attention should be paid to cross-gender and gender-specific care needs of AYAs (Görres et al. 2024). Therapies designed to enhance sleep could evaluate psychological elements such as anxiety and depression, in addition to quality of life, in order to facilitate comprehensive treatment. Evidence-based approaches to sleep disturbances, including cognitive-behavioral therapy for insomnia (CBT-I; Hinterberger et al. 2024), should be investigated further, particularly in AYA patients. Mindfulness-based and other mind–body approaches may also be relevant; however, their use was not assessed in the present study, and no conclusions regarding their association with sleep quality can therefore be drawn from our data.

Considering the potential adverse effects of sleep medication, non-pharmacological approaches and lifestyle modifications to enhance sleep may be considered in the management of sleep disturbances in AYA patients. These may include maintaining consistent sleep and wake times and other sleep hygiene strategies. However, the present study did not assess the use or effectiveness of specific sleep-promoting strategies, and no conclusions regarding their effects on sleep quality can therefore be drawn from our findings.

The association between unmet needs related to energy and tiredness and poorer sleep quality suggests that fatigue-related concerns should be considered when assessing and managing sleep disturbances in AYA patients.

## Conclusion

Sleep disturbances in AYA patients remain an understudied but clinically relevant aspect of cancer survivorship care. The observed associations of poorer sleep quality with psychological distress, lower functional quality of life, and unmet needs related to lack of energy and tiredness suggest that sleep disturbances should be considered within the broader psychosocial context of AYA survivorship. Routine assessment of sleep disturbances may help identify patients who require further evaluation or support. Further longitudinal research is needed to clarify the direction of these associations, investigate underlying mechanisms and identify appropriate strategies for the prevention and management of sleep disturbances in AYA patients.

## Data Availability

The data of this study are available on request from the corresponding author. The data are not publicly available due to privacy or ethical restrictions.
